# Phytoplankton in Lakes of Different Nutrient Levels in the Middle Yangtze River: Annual Variations in Community Structure and Key Driving Factors

**DOI:** 10.3390/plants15142108

**Published:** 2026-07-08

**Authors:** Xing Wang, Xiaodong Wu, Xuejian Cai, Jingjing Feng, Jinglei Peng, Xuguang Ge, Fan Xun, Xinhui Yu, Haoyue Li, Ziru Pan, Xin Mou, Shilu Ye

**Affiliations:** 1 College of Urban and Environmental Sciences, Hubei Normal University, Huangshi 435002, China; 2024x07051106@stu.hbnu.edu.cn (X.W.); gxg76@hbnu.edu.cn (X.G.); xunfan130@163.com (F.X.); yuxinhui_1998@163.com (X.Y.); 2024x07051108@stu.hbnu.edu.cn (H.L.); 2025x07051108@stu.hbnu.edu.cn (Z.P.); muxin021126@163.com (X.M.); 19737752861@163.com (S.Y.); 2 Wanghu Lake Wetland Ecosystem Field Scientific Observation and Research Station, Hubei Normal University, Huangshi 435002, China; 3 Huangshi Key Laboratory of Soil Pollution and Control, Hubei Normal University, Huangshi 435002, China; 4 Huangshi Chengfa Environmental Testing Technology Co., Ltd., Huangshi 435002, China; alpsjian@163.com (X.C.); fjj2026888@163.com (J.F.); p981189271@163.com (J.P.)

**Keywords:** lakes, phytoplankton, functional groups, growth strategies

## Abstract

Phytoplankton communities are highly sensitive to environmental change and serve as key indicators of lake ecosystem health. In this study, four typical lakes in the middle Yangtze River (Xiandao, Bao’an, Wanghu, and Cihu Lake) were investigated through field monitoring to reveal the spatiotemporal patterns of phytoplankton communities and their driving environmental factors. Significant seasonal variation and spatial heterogeneity were observed. Although species composition varied, Chlorophyta, Cyanobacteria, and Bacillariophyta were dominant in all lakes. In the eutrophic Bao’an, Cihu, and Wanghu lakes, Cyanobacteria dominated the annual average abundances. Based on Reynolds’ functional group (FG) classification, 27 groups were identified. Dominant FG had seasonal successions. Canonical Correspondence and redundancy analyses indicated that water transparency, suspended solids, phosphate, and water temperature were the key environmental factors driving phytoplankton community succession. This study elucidated the response patterns of phytoplankton communities in lakes with varying nutrient levels to seasonal changes as well as the mechanisms underlying shifts in their growth strategies, thereby providing a theoretical framework for the ecological restoration of eutrophic lakes.

## 1. Introduction

As the main source of initial productivity in aqueous ecosystems [1], phytoplankton play a vital role in the material cycle, energy flow, and information transfer within lake ecosystems. Because of their rich species diversity, small cell size, and short growth cycle, phytoplankton are highly susceptible to environmental changes, such as temperature, light, and nutrient availability, which can affect their biomass, composition, and diversity [2]. Consequently, phytoplankton are key indicators of lake ecosystem responses to environmental change and play a fundamental role in maintaining ecosystem function. Excessive nutrient levels in lakes can alter the dissolved oxygen levels, increase algal biomass, and adversely affect the structure of aquatic communities, food webs, and carbon and nutrient cycling [3].

Traditional taxonomic approaches fail to comprehensively capture the complex relationships between phytoplankton and their environment. In contrast, functional group (FG) classification considers both species homology and functional specificity, grouping phytoplankton adapted to specific habitat characteristics and sharing similar sensitivities into the same FGs, thereby characterizing phytoplankton responses to environmental factors relatively accurately [4,5,6]. Currently, research on lake phytoplankton is well-established both domestically and internationally, with most studies primarily focusing on community structures, spatial distribution patterns, and influencing factors in large natural lakes (such as Lake Tai [7], Poyang Lake [8], Chaohu Lake [9], and Dongting Lake [10]). Existing research indicates that the dominant environmental factors governing lake phytoplankton community structure exhibit marked regional and seasonal variations: at the regional scale, phytoplankton communities in alpine lakes are primarily driven by local environmental factors such as total phosphorus (TP), with diffusion constraints playing a negligible role [11]. On a seasonal scale, FGs in subtropical lakes display distinct differences between the rainy and dry seasons, with water temperature, precipitation, and nutrients being the dominant factors [12]. Furthermore, phytoplankton in lakes with extreme environmental conditions (such as high salinity, elevated alkalinity, or low pH) are often dominated by a single species, and the community structure tends to be simplified [13]. There remains considerable scope for further investigation of the key environmental factors influencing phytoplankton community structure and abundance under different regional and seasonal conditions.

This study focused on four typical lakes in the middle reaches of the Yangtze River: Xiandao Lake, Bao’an Lake, Wanghu Lake, and Cihu Lake. By integrating techniques such as FG classification, multivariate statistical analysis, and FG ecological strategy analysis, we systematically investigated the seasonal succession patterns and key driving mechanisms of the phytoplankton community structure in lakes with varying nutrient levels. While deepening our theoretical understanding, this study elucidated systematic shifts in the assembly strategies of phytoplankton FGs, revealing responsive patterns of lake phytoplankton communities to changes in nutrient levels and providing practical guidance for the precise regulation and ecological restoration of eutrophic lakes. The specific research objectives were to: (1) reveal seasonal succession patterns and key environmental drivers of phytoplankton community structure in lakes with different nutrient levels, and (2) elucidate the mechanisms underlying shifts in phytoplankton FG assembly strategies along nutrient gradients and their ecological significance.

## 2. Results

### 2.1. Chemical and Physical Properties of Water

For variables that satisfied the normality and homoscedasticity assumptions, one-way analysis of variance was used to compare differences in environmental factors among the four lakes and across seasons. The four lakes in the middle Yangtze River exhibited significant spatiotemporal variability in physicochemical and nutrient parameters (Table 1). Xiandao Lake had a significantly higher average annual water transparency than the other lakes (*p* < 0.05), averaging 3.41 m, and its permanganate index (COD_Mn_) and suspended solid (SS) values were significantly lower than those of the other three lakes (*p* < 0.05). Regarding nutrient indicators, Wanghu and Cihu exhibited higher average total nitrogen (TN) concentrations. Furthermore, the average concentrations of TP, phosphate (PO_4_^3−^-P), and ammonia nitrogen (NH_3_-N) in Wanghu Lake were higher than those in the other three lakes (*p* < 0.05). The spatial pattern of nitrate (NO_3_^−^-N) concentrations differed from that of other nutrients; Wanghu Lake had the lowest average NO_3_^−^-N concentration at 0.07 mg/L, whilst Cihu Lake had the highest at 0.22 mg/L *(p* < 0.05). The annual average Trophic Level Index (TLI) for Cihu Lake was 57.13, and the TLI values for Bao’an and Wanghu Lake were slightly lower, with annual averages of 55.40 and 54.79, respectively, indicating that all three lakes were in a state of mild eutrophication. Lake Xiandao exhibited the lowest TLI and was oligotrophic.

### 2.2. Phytoplankton Community Structure and Dominant Species

**Figure 1:**
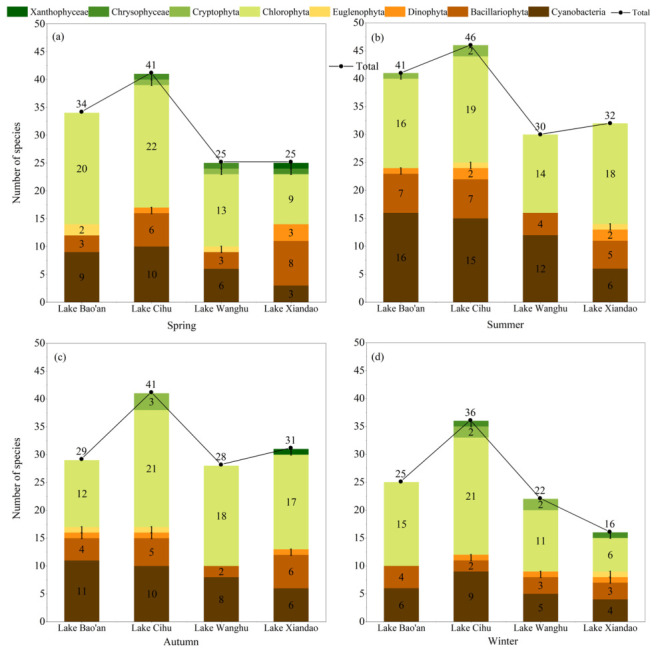
(**a**–**d**) Phytoplankton species composition in a typical lake in the middle Yangtze River.

Between October 2022 and August 2023, 183 species belonging to eight phyla of phytoplankton were identified in four representative lakes in the middle reaches of the Yangtze River (Table S1). Of these, the phylum Chlorophyta was the most abundant, comprising 79 species, followed by 40 species of Bacillariophyta, 34 of Cyanobacteria, 13 of Euglenophyta, and 8 of Dinophyta. Species richness was relatively low for Cryptophyta, Chrysophyta, and Xanthophyta, with 4, 3, and 2, respectively. Bao’an Lake had the highest phytoplankton species richness with 140 species identified, Cihu Lake had 111 species, Wanghu Lake had the lowest diversity with 98 species identified, and Xiandao Lake had 102 species (Figure 1). The composition of phytoplankton species differed between the lakes; however, all were dominated by Chlorophyta, Cyanobacteria, and Bacillariophyta (Figure 1). Phytoplankton species richness in all four lakes peaked in summer, with Chlorophyta exhibiting the highest richness, followed by Cyanobacteria. Except for Xiandao Lake, species richness followed a consistent seasonal pattern, decreasing in the order of summer, spring, winter, and autumn. In Xiandao Lake, species richness was lowest in winter.

Bao’an, Cihu, Wanghu, and Xiandao contained 17, 18, 18, and 17 dominant species, respectively (Table 2). Based on the principle that dominant species are those with a dominance index Y ≥ 0.02, Cyanobacteria predominated among the dominant phytoplankton species in Bao’an Lake, with *Limnothrix* sp., *Pseudoanabaena limnetica* (Lemmermann) Komárek & J.Komárková-Legnerová, and *Aphanizomenon* sp. being the main species. Among the Bacillariophyta, Cyclotella sp. became the dominant species in winter and spring, whereas among the Chlorophyta, *Scenedesmus bijuga* (Turpin) Lagerheim was frequently identified in other lakes. Cihu Lake is home to 18 dominant species, with the phylum Cyanobacteria being particularly well-represented, and the most frequently occurring species being *Limnothrix* sp., *P. limnetica*, and *Merismopedia minima* Gomont. Wanghu Lake contained 18 dominant species. Cyanobacteria, such as *Dolichospermum* sp., *Oscillatoria tenuis* C.Agardh ex Gomont, and *Microcystis* sp., were the dominant species across all four quarters. Xiandao Lake had 17 dominant species, including eight belonging to the phylum Cyanobacteria. Among these, *Dactylococcopsis* sp. exhibited the highest dominance in summer, whereas *Dolichospermum convolutus* (P.G.Richter) Wacklin, L.Hoffmann & Komárek was dominant in winter. There were three species of Bacillariophyta, four species of Chlorophyta, and one each of Chrysophyceae and Xanthophyceae, with a dominance index of *Botrydiopsis* sp. of 0.48, making them the most dominant species in spring. *Cyclotella* sp. and *Limnothrix* sp. were identified in all four lakes. In terms of species richness, Chlorophyta was the most diverse phylum across all lakes; in terms of cell abundance, Cyanobacteria dominated the three eutrophic lakes on an annual average.

### 2.3. Composition of Phytoplankton FGs and Distribution Characteristics of Dominant Functional Groups

**Figure 2:**
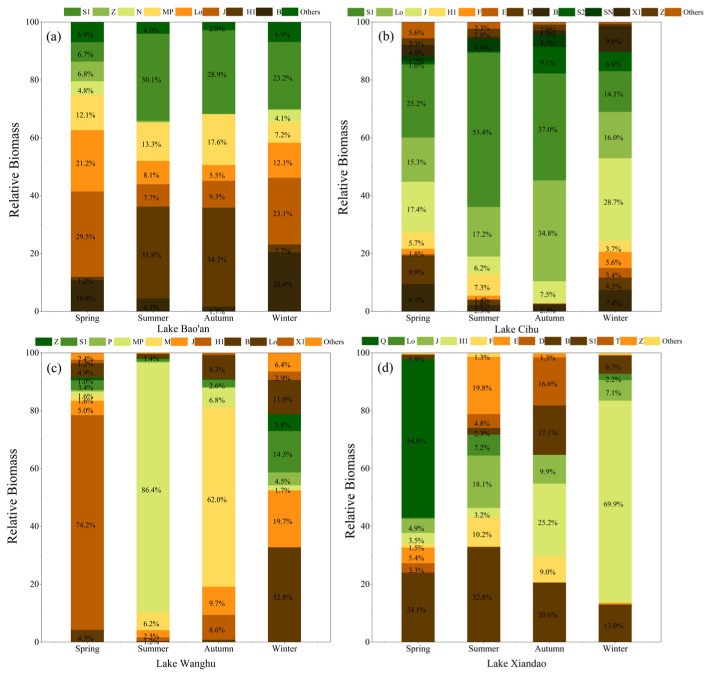
Relative biomass of phytoplankton functional groups (FGs) in typical lakes of the middle Yangtze River. Panels (**a**–**d**) correspond to Bao’an Lake, Cihu Lake, Wanghu Lake and Xiandao Lake, respectively. All FG letter codes and their associated representative species, habitat characteristics and growth strategies are fully defined in Table 3. Only percentage labels with values greater than 1 are displayed in the figure. All percentage values are rounded to one decimal place; therefore, the sum of the percentages may not equal exactly 100%.

Based on the Reynolds FG classification, 27 FGs were identified across the four lakes; their habitat characteristics and growth strategies are defined in Table 3. FGs with relative biomass >5% were defined as dominant groups, and their seasonal dynamics are shown in Figure 2. In Bao’an Lake, spring assemblages were dominated by generalist taxa adapted to well-mixed shallow waters (groups J, Lo, MP, and B). The community shifted to nitrogen-fixing and low-light-tolerant cyanobacteria (groups H1, S1, and MP) in summer and autumn, returned to a diatom-Chlorophyta assemblage (groups B, J, and S1) in winter, and maintained persistent cyanobacterial dominance throughout the year, with shade-tolerant filamentous cyanobacteria and generalist picoplankton (groups S1 and Lo) as core components. Chlorophyta and small diatoms increased in proportion in winter. Wanghu Lake showed distinct seasonal turnover: nitrogen-fixing cyanobacteria (group H1) dominated in spring, disturbance-tolerant turbid-water taxa (group MP) prevailed in summer, and bloom-forming colonial cyanobacteria (group M) became dominant in autumn. The winter assemblage shifted back to a mixed diatom-Chlorophyta community (groups B, J, S1, and Lo). In the oligotrophic Xiandao Lake, mixotrophic flagellates and small diatoms (groups Q and B) dominated in spring, oligotrophic-adapted diatoms and picocyanobacteria (groups B and Z) prevailed in summer, and nitrogen-fixing cyanobacteria (groups H1 and B) were dominant in autumn and winter. Overall, the dominant FGs shifted from stress-tolerant S-strategy taxa in the oligotrophic lake to fast-growing R-strategy taxa in the eutrophic lakes along the nutrient gradient.

### 2.4. Phytoplankton Abundance and Biomass

**Figure 3:**
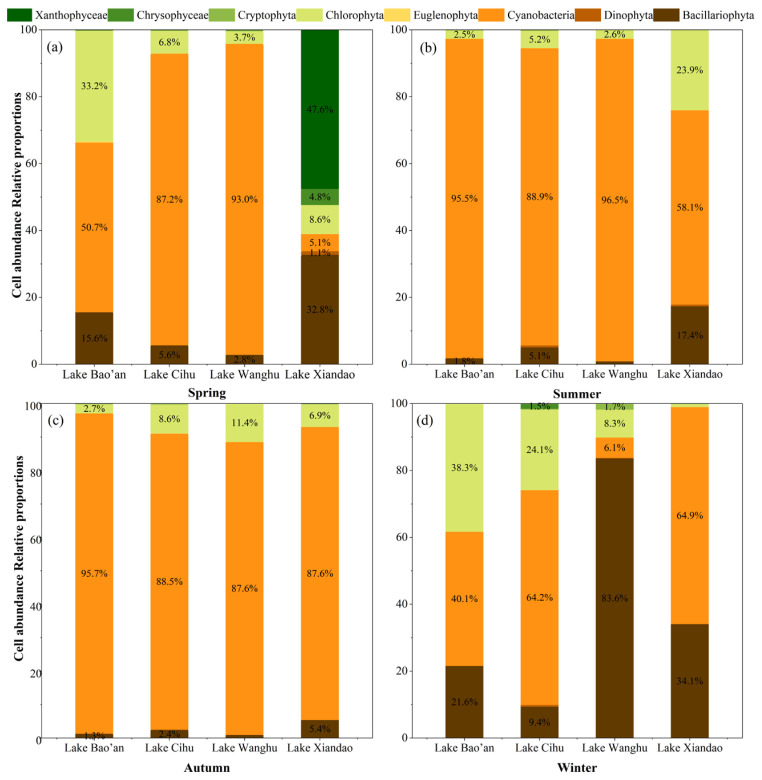
(**a**–**d**) Abundance of phytoplankton taxa in typical lakes of the middle Yangtze River, Percentage abundance of phytoplankton taxa (horizontal axes represent lakes). Only percentage labels with values greater than 1 are displayed in the figure. All percentage values are rounded to one decimal place; therefore, the sum of the percentages may not equal exactly 100%.

In terms of spatial distribution, phytoplankton abundance was significantly higher in Cihu Lake than in Xiandao Lake (*p* < 0.05), whereas there was no significant difference in phytoplankton abundance between Bao’an Lake and Wanghu Lake. In terms of the annual average abundance, Cyanobacteria ranked first among the phytoplankton groups in all lakes (Figure 3). The total phytoplankton abundance in Bao’an Lake reached a maximum of 598.33 × 10^6^ cells/L, and Cyanobacteria accounted for >90% of the total phytoplankton, with absolute dominance. Cyanobacteria and Chlorophyta were predominant during winter and spring. In Cihu Lake, Cyanobacteria dominated the phytoplankton abundance throughout all four seasons, accounting for an annual average of 82%. In Wanghu Lake, Cyanobacteria were predominant in all seasons except winter, when Bacillariophyta accounted for 84% of the total abundance. In Xiandao Lake, Xanthophyceae were dominant only in spring (47.6%), whereas Cyanobacteria remained the most abundant in all other seasons.

**Figure 4:**
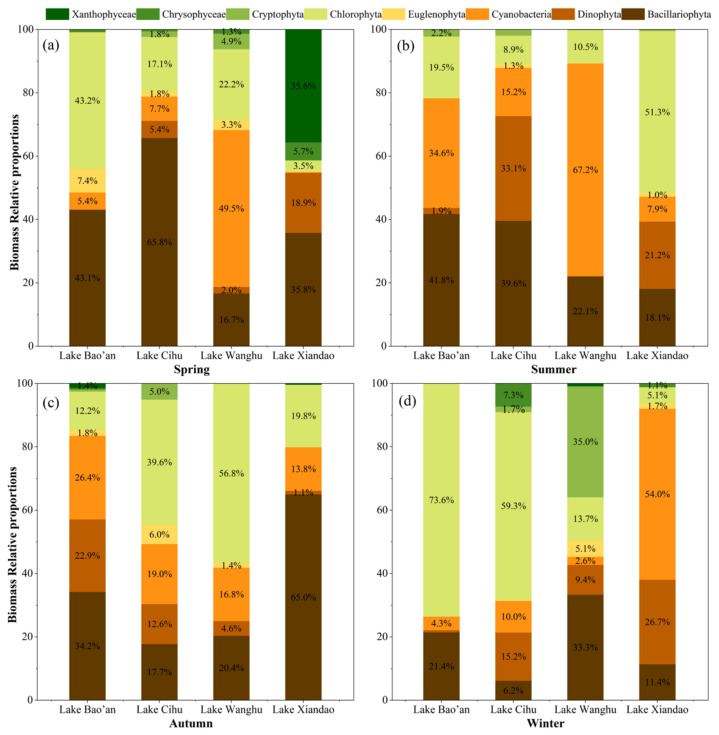
(**a**–**d**) Biomass of phytoplankton taxa in typical lakes of the middle Yangtze River biomass of phytoplankton taxa (horizontal axes represent lakes).Only percentage labels with values greater than 1 are displayed in the figure. All percentage values are rounded to one decimal place; therefore, the sum of the percentages may not equal exactly 100%.

Cihu Lake had the highest annual average biomass, followed by Bao’an and Wanghu Lake, whereas Xiandao Lake had the lowest. In Bao’an Lake, Cyanobacteria and Bacillariophyta jointly dominated during the summer and autumn, whereas Chlorophyta predominated during the winter and spring (Figure 4). In Cihu Lake, Bacillariophyta and Dinophyta dominated the biomass in spring and summer, accounting for more than 70% of the total biomass, and the proportion of Chlorophyta increased significantly in autumn and winter. The biomass in Wanghu Lake exhibited distinct seasonal succession: Cyanobacteria and Bacillariophyta accounted for a high proportion in spring and summer, followed by Chlorophyta in autumn, and Cryptophyta became the dominant group in winter. The biomass composition in Xiandao Lake exhibited distinct seasonal variations: in spring, Xanthophyceae and Bacillariophyta jointly dominated; in summer, Chlorophyta accounted for the highest proportion; in autumn, Bacillariophyta became dominant; and in winter, Cyanobacteria and Dinophyta jointly dominated.

### 2.5. Phytoplankton Diversity Index

**Figure 5:**
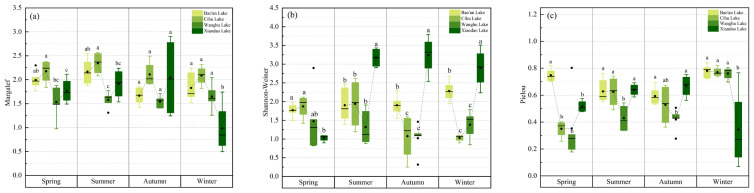
Phytoplankton diversity indices of the four studied lakes. Panels (**a**–**c**) correspond to the Margalef richness index, Shannon–Wiener diversity index and Pielou evenness index, respectively. The x-axis presents four lakes in sequence: Bao’an Lake, Cihu Lake, Wanghu Lake and Xiandao Lake. Different lowercase letters indicate significant differences among lakes (*p* < 0.05). The standardized evaluation thresholds are 1 for the Margalef index, 1 for the Shannon–Wiener index, and 0.3 for the Pielou index.

Xiandao Lake exhibited the highest annual averages for Shannon–Wiener diversity, Margalef richness, and Pielou evenness indices. Furthermore, significant seasonal variations were observed in Xiandao Lake, with consistently higher values in summer and autumn than in winter and spring (Figure 5). According to the one-way analysis of variance results, the Shannon-Weiner indices for Bao’an, Cihu, and Wanghu lakes differed significantly from those of Xiandao Lake (*p* < 0.05), whereas no significant differences were observed in the Margalef richness or Pielou evenness indices among the lakes. The Shannon-Wiener index for Xiandao Lake indicated light pollution, whereas all other lakes exhibited moderate pollution levels during the survey period. The Margalef richness index for phytoplankton in Wanghu Lake indicated a severe pollution level, whereas the other lakes had moderate pollution levels. The average Pielou uniformity index for Xiandao Lake in spring, summer, and autumn was >0.5, indicating mild pollution, whereas the remaining lakes exhibited moderate pollution.

### 2.6. Major Environmental Factors Influencing the Structure of Phytoplankton Communities

**Figure 6:**
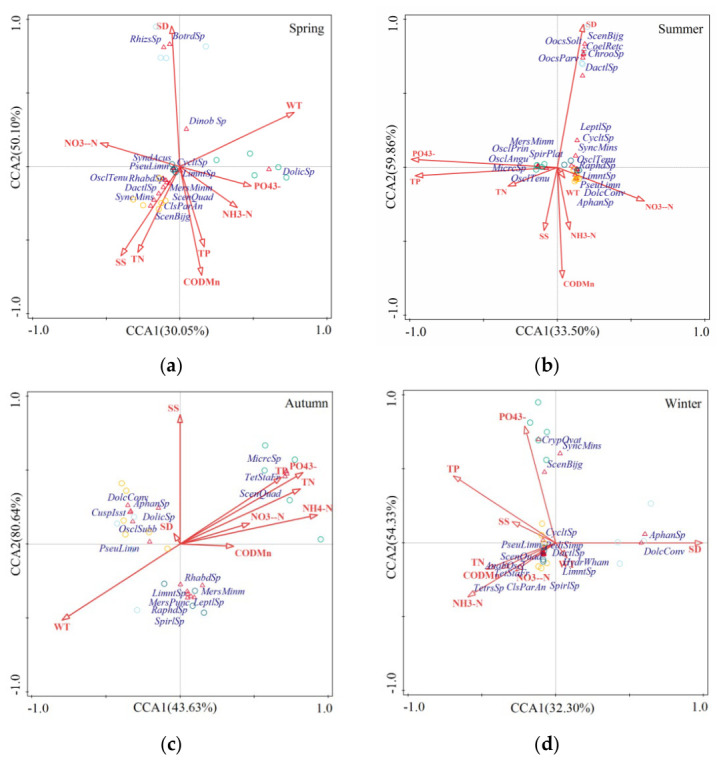
Canonical correspondence analysis (CCA) of dominant phytoplankton species and environmental factors. (**a**) Spring; (**b**) Summer; (**c**) Autumn; (**d**) Winter. Red arrows indicate water physicochemical parameters (WT: water temperature; SD: Secchi depth; SS: suspended solids; TN: total nitrogen; TP: total phosphorus; NO_3_^−^-N: nitrate nitrogen; PO_4_^3−^-P: phosphate phosphorus; NH_3_-N: ammonia nitrogen; COD_Mn_: permanganate index); triangles represent dominant phytoplankton species (dominant species are defined by dominance index Y > 0.02 and detailed in Table 2); circles represent sampling sites (yellow: Bao’an Lake; dark blue: Cihu Lake; green: Wanghu Lake; light blue: Xiandao Lake). Detrended correspondence analysis (DCA) was performed prior to ordination; as the gradient length of the first DCA axis was >4, a unimodal model (CCA) was applied.

Seasonal shifts in phytoplankton community structure across the four lakes were regulated by divergent sets of environmental drivers (Figure 6). Canonical correspondence analysis (CCA) ordination demonstrates that spring community variation was primarily associated with transparency (SD), NO_3_^−^-N, and water temperature (WT); summer variation with SD, SS, and PO_4_^3−^-P; autumn variation predominantly with WT and SS; and winter variation with SD, SS, and PO_4_^3−^-P. Nitrogen is the primary environmental driver of the seasonal dynamics in phytoplankton communities in Bao’an Lake and Cihu Lake. In contrast, phosphorus exerts a more significant regulatory influence on community formation in Wanghu Lake, whereas water depth exerts continuous and dominant control over seasonal community succession throughout the annual cycle in Xiandao Lake.

**Figure 7:**
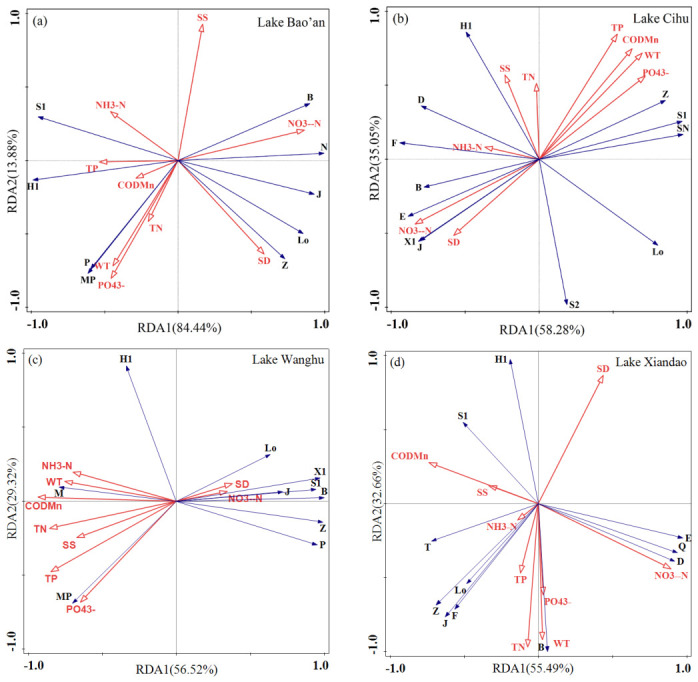
Redundancy analysis (RDA) of dominant phytoplankton functional group biomass and environmental factors. (**a**) Bao’an Lake; (**b**) Cihu Lake; (**c**) Wanghu Lake; (**d**) Xiandao Lake. Red arrows indicate water physicochemical parameters (WT: water temperature; SD: Secchi depth; SS: suspended solids; TN: total nitrogen; TP: total phosphorus; NO_3_^−^-N: nitrate nitrogen; PO_4_^3−^-P: phosphate phosphorus; NH_3_-N: ammonia nitrogen; COD_Mn_: permanganate index); blue arrows represent dominant phytoplankton functional groups (functional group codes are defined in Table 3). Detrended correspondence analysis (DCA) was performed prior to ordination; as the gradient length of the first DCA axis was <3, a linear model (RDA) was applied.

Redundancy analysis (RDA) was employed in subsequent ordination analyses to investigate the relationships between dominant FGs and environmental factors. The RDA results indicated that environmental factors explained 84.44% and 13.88% of the variance in the first two axes of the dominant FGs in Bao’an Lake, respectively (Figure 7). SS, SD, PO_4_^3−^-P, and NO_3_^−^-N were identified as the primary environmental factors influencing the seasonal variation of functional communities in Bao’an Lake. FGs B, N, J, Lo, and Z exhibited positive correlations with NO_3_–N and SD, whereas FGs S1, H1, P, and MP showed negative correlations with these factors. FGs S1 and H1 were positively correlated with NH_3_-N, TP, and COD_Mn_, whilst FGs P and MP showed strong positive correlations with PO_4_^3−^-P, TN, and WT.

## 3. Discussion

### 3.1. Analysis of Structural Characteristics of Phytoplankton Communities

In the four lakes in the middle Yangtze River, Cyanobacteria were the dominant phytoplankton species in most seasons, followed by Chlorophyta and Bacillariophyta, which is broadly consistent with previous findings from studies involving Tai Lake and Chaohu Lake [14,15,16,17]. Except for Xiandao Lake, where Xanthophyceae constituted the largest proportion of phytoplankton abundance in spring, Cyanobacteria were the most abundant in the other three lakes. Cyanobacteria were the dominant phytoplankton species in the Wanghu, Cihu, and Bao’an lakes, whereas Bacillariophyta and Chlorophyta were predominant in the Xiandao Lake. Dinophyta, Bacillariophyta, and Cryptophyta are commonly found in mesotrophic lakes, whereas Cyanobacteria and Chlorophyta are pollution- and heat-tolerant phytoplankton, respectively. Summer rainfall causes large amounts of nitrogen and phosphorus to enter lakes, creating conditions favorable for the growth of thermophilic cyanophytes and chlorophytes, which are often regarded as indicators of water eutrophication [18]. In contrast, Xiandao Lake exhibited higher summer Dinophyta biomass, possibly because it is a deep-water lake with a relatively low WT and high dissolved oxygen (DO) concentrations.

The area surrounding Bao’an Lake contains many fishponds and cultivated fields, with relatively little land designated for construction [19]. The summer phytoplankton community was dominated by the phyla Chlorophyta, Cyanobacteria, and Bacillariophyta, with a species composition characterized by a Chlorophyta–Cyanobacteria–Bacillariophyta pattern, whereas phytoplankton abundance followed a Cyanobacteria-Chlorophyta-Bacillariophyta pattern. This indicates that, outside of summer and autumn Cyanobacteria bloom periods, Bacillariophyta play a significant role in terms of biomass, suggesting that the lake may experience a brief ‘Bacillariophyta window period,’ as Bacillariophyta are often considered indicators of good water quality [20]. The phytoplankton community structures of the Cihu and Wanghu Lakes were of the Cyanobacteria–Chlorophyta type, with water bodies generally classified as moderately to heavily polluted. The main source of pollution in Cihu Lake is improper discharge of domestic sewage [21]. Wanghu Lake and its surrounding areas are characterized by extensive farmland and fishponds. Improper discharge of aquaculture effluents and inflow of agricultural wastewater have led to reduced water transparency and elevated nitrogen and phosphorus concentrations, resulting in the deterioration of water quality. Furthermore, the barrier effect of impervious surfaces on construction land and in urban areas surrounding lakes results in the discharge of untreated mixed rainwater and sewage into lakes (and their tributaries), leading to eutrophication [22]. Additionally, Cihu and Wanghu Lakes have poor water exchange capacities with external water bodies [23], making it difficult to alter the nutrient status and providing favorable conditions for the mass proliferation of certain pollution-tolerant Cyanobacteria and Chlorophyta species [24,25].

Community diversity indices are commonly used to evaluate the stability of a biological community’s structure; the greater the number of species within a community, the stronger its negative feedback mechanisms, and the more stable the ecosystem tends to be [26,27]. The mean Shannon-Wiener and Pielou indices for Xiandao Lake were both >1.030, and these values were markedly higher than those for the other lakes (*p* < 0.05). It may be attributed to the fact that Xiandao Lake is less affected by human activity and has a higher vegetation cover, and a rich diversity of submerged vegetation, which are conducive to the growth of various types of phytoplankton. In contrast, the Pielou indices for Cihu and Wanghu lakes were relatively low, indicating low community diversity. Because of endogenous and exogenous pollution, large amounts of nutrients enter water bodies, leading to continuous deterioration in water quality, a finding consistent with the results of the physicochemical monitoring data.

### 3.2. Relationship Between Dominant Phytoplankton Species and Environmental Factors

According to the CCA ordination diagram, WT, SD, SS, NO_3_^−^-N, and PO_4_^3−^-P were the primary vectors influencing the species composition of phytoplankton in various lakes (Figure 6). Water temperature is one of the environmental factors with the greatest direct effect on phytoplankton growth. Rising WTs can increase enzyme activity within phytoplankton cells and accelerate photosynthesis, thereby promoting growth and reproduction. Different algal species have distinct optimal temperature ranges. A sustained rise in WT often favors thermophilic Cyanobacteria and certain Chlorophyta, allowing them to become dominant species, while suppressing groups such as Bacillariophyta, which prefer lower WTs. The CCA results also showed that the WT was positively correlated with most phytoplankton groups, thus supporting the above conclusion. The effect of SD on phytoplankton was primarily reflected by light availability. Considering Xiandao Lake as an example, the dominant phytoplankton species were positively correlated with the SD. An increase in the SD enhances the depth of water penetration by light, providing phytoplankton with more abundant light energy, thereby promoting photosynthesis and increasing phytoplankton abundance. Notably, the massive proliferation of phytoplankton can reduce SD, thus creating a dynamic, reciprocal relationship between the two. SS is a key factor regulating phytoplankton biomass, and during turbid phases (total suspended solids > 60 mg/L), phytoplankton biomass remains high despite limited light. The ratio of specific nitrogen forms can drive the succession of dominant species towards Cyanobacteria (such as *Microcystis* sp. and *Oscillatoria* sp.), and high SS environments may interfere with the feeding of filter-feeding zooplankton (such as large copepods), reducing downward control on phytoplankton. This reveals that under ample nutrient conditions, SS indirectly influences the phytoplankton community structure by altering light conditions and biological interactions; its positive effect via nutrient transport may outweigh the negative effects of shading [28,29].

The driving roles of the nutrients exhibited marked differences across the lakes. In Wanghu Lake, where Cyanobacteria and Chlorophyta were the dominant groups, phytoplankton abundance was positively correlated with PO_4_^3−^P and TP, indicating that the dominant phytoplankton species in this lake may prefer high-phosphorus environments. Although moderate increases in phosphorus can promote phytoplankton growth, excessively high P concentrations can trigger eutrophication. Recent studies reveal that Cyanobacteria can substitute non-phospholipids for phospholipids, thereby reducing the cellular demand for P, maintaining a competitive advantage, and even forming blooms under low phosphorus stress [30]. In contrast, in Cihu and Bao’an Lake, where Cyanobacteria, Bacillariophyta, and Chlorophyta jointly dominated, phytoplankton abundance was positively correlated with nitrogen levels, SS, and COD_Mn_, but negatively correlated with phosphorus, indicating that these groups are better adapted to high-nitrogen, eutrophic water environments. Phytoplankton growth depends on both nitrogen and phosphorus; however, the limiting effects of these two elements often vary with the environment, and the nitrogen-to-phosphorus ratio, rather than the absolute concentration, is a key factor determining biomass [31]. In water bodies where phosphorus is relatively abundant but nitrogen is limited, some cyanobacteria (such as *Chlorella* sp.) can alleviate nitrogen limitation through nitrogen fixation, a process regulated by phosphorus availability. Relevant studies have shown that the phytoplankton trypsin PtTryp2 can respond to nutrient fluctuations by regulating gene expression, thereby maintaining homeostasis of the intracellular nitrogen-to-phosphorus stoichiometric ratio [32]. Furthermore, the decomposition of readily degradable organic matter, represented by COD_Mn_, links organic matter and inorganic nutrient pools. In summary, there were marked differences in the environmental factors governing phytoplankton community structure across different seasons. WT, SD, nutrients, and SS collectively influenced phytoplankton growth, competition, and succession, reflecting the complexity of multi-factor coupling within lake ecosystems.

### 3.3. Relationship Between Seasonal Variation Characteristics of Dominant Phytoplankton FGs and Environmental Factors

During the summer and autumn seasons, the H1 FGs, represented by *Anabaena* sp., adapted to eutrophication, tolerated water stratification and low-nitrogen environments, and exhibited a marked preference for strong light conditions. In spring, nutrient release from bottom sediments into the water decreases, whereas heavy rainfall in early summer dilutes the water body, resulting in a significant short-term reduction in nitrogen concentrations in Bao’an Lake [33]. Compared to FGs such as S1, J, and Lo, H1 exhibited a faster growth rate under low-nitrogen conditions, thereby becoming the dominant FG in Bao’an Lake during summer and autumn. The RDA plot for Cihu Lake (RDA1 explanatory power 58.28%, RDA2 explanatory power 35.05%) showed that the S1, Z, and SN FGs, located on the positive axis of the first principal component, were positively correlated with TP, PO_4_^3−^-P, WT, and COD_Mn_, indicating that these FGs are dependent on a high phosphorus load environment. Throughout the four seasons, the FGs in Cihu Lake were dominated by S1 and Lo (in summer, S1 and Lo accounted for 53.33% and 17.14%, respectively). This persistent S1/Lo dominance pattern indicated that Cihu Lake is in a Cyanobacteria-dominated turbid-water steady state [34]. The S1 and Lo groups were primarily composed of Cyanobacteria and Chlorophyta, which thrived in relatively warm WTs; consequently, these FGs became dominant in summer. The resurgence of group J in winter was closely linked to the recovery of Chlorophyta under low-temperature and high-SD conditions. Although the Lo group showed no significant correlation with any single environmental factor, it remained dominant throughout the year, classifying it as a ‘tolerant’ FG.

The RDA plot for Wanghu Lake (RDA1 explanatory power 56.52%, RDA2 explanatory power 29.32%) revealed that the seasonal succession of FGs in Wanghu Lake exhibited a typical R-strategy-dominated pattern. The MP group dominated under high-light and strong-mixing conditions in summer, whereas the M group became dominant when water stability returned in autumn. This succession pattern was consistent with the findings of Paerl et al. [35,36]. The MP and M groups were positively correlated with SS and negatively correlated with SD, reflecting their adaptation to environments with low light and high SS [37]. During the summer rainy season, water-column stability is disrupted, leading to turbidity and creating favorable conditions for MP FGs to grow rapidly and become the dominant FGs. In autumn, the dominant FGs, M, primarily consisting of *Microcystis* sp., proliferated extensively, which was associated with more severe eutrophication resulting from large-scale fertilizer-based aquaculture in Wanghu Lake. The RDA plot for Xiandao Lake (RDA1 explanatory power 55.49%, RDA2 explanatory power 32.66%) indicated that the seasonal succession of FGs followed an S-strategy-dominated pattern: in spring, Group Q (*Gonyostomum* sp., 55%) was dominant; in summer, Groups B (*Cyclotella* sp., 33%) and Z (*Synechocystis minuscula*, 20%) were dominant; and in autumn and winter, Group H1 (25% and 70%) was dominant. The dominant species, Group H1, was positively correlated with SD, indicating its dependence on high transparency and light conditions, which explains why these FGs were dominant in autumn and winter. The absolute dominance of the Q FGs in spring confirms the competitive advantage of mixotrophic FGs in oligotrophic waters [38,39], whilst the Z group is capable of tolerating high-transparency, low-nutrient waters; its extremely small cell size (<2 μm) reduces sedimentation loss and facilitates the efficient utilization of low-concentration nutrients [40]. Group B was adapted to fully mixed, mesotrophic, small- to medium-sized water bodies.

Differences in the RDA ordination axis structures and relationships between environmental factors and FGs across the four lakes revealed systematic shifts in the mechanisms governing phytoplankton community assembly along nutrient gradients. Xiandao Lake (oligotrophic) was dominated by S-strategy FGs (Q, B, and Z), which reflect the diversity of survival strategies in a low-resource environment. Cihu and Wanghu lakes (eutrophic) were dominated by R-strategy FGs (S1, MP, and M), reflecting rapid growth strategies in high-resource, high-disturbance environments. Bao’an Lake (transitional) exhibits an annual alternation between the S- and R-strategies, reflecting fluctuations in the system between the two steady states. From a lake management perspective, the findings of this study provide strategies for the ecological restoration of lakes with varying trophic statuses. For eutrophic lakes (Cihu Lake and Wanghu Lake), the focus should be on controlling external nitrogen and phosphorus inputs; for transitional lakes (Bao’an Lake), interventions should be implemented during the window period (April–May) when the system transitions from a clear-water steady state in spring to a turbid-water steady state in summer and autumn; for oligotrophic lakes (Xiandao Lake), efforts should focus on maintaining high transparency and low nutrient levels, whilst remaining vigilant against the risk of reduced FGs diversity caused by external pollution inputs.

## 4. Materials and Methods

### 4.1. Study Area

**Figure 8:**
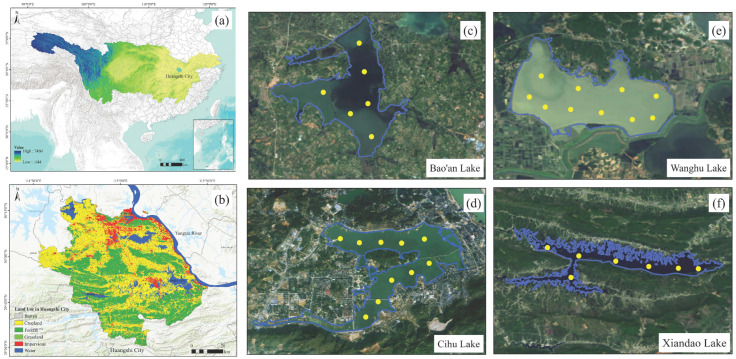
Study area located in Hubei Province, China. (**a**) the south-eastern Hubei region, (**b**) land use in Huangshi City, (**c**) Bao’an Lake, (**d**) Cihu Lake, (**e**) Wanghu Lake, (**f**) Xiandao Lake.

In this study, we selected four lakes in the middle reaches of the Yangtze River—Cihu Lake, Xiandao Lake, Wanghu Lake, and Bao’an Lake—as the subjects of investigation; these four lakes exhibit varying nutrient statuses. To ensure full lake representativeness, sampling sites were arranged covering the central lake area, nearshore zones, and inlet/outlet areas of each lake, in accordance with lake morphology, surface area, and habitat heterogeneity. Four seasonal sampling events were conducted in October 2022 (autumn), January 2023 (winter), May 2023 (spring), and August 2023 (summer), covering the entire water area of all four studied lakes (Figure 8). Cihu Lake (30°12′30″ N, 115°03′01″ E) is situated in the center of Huangshi City and is the city’s largest urban lake. It has an average depth of 2.7 m, a catchment area of 62.20 km^2^, and a surface area of 10.5 km^2^. Wanghu Lake (29°52′01″ N, 115°20′02″ E) is situated in the north-east of Yangxin County, Huangshi, within the middle and lower reaches of the Yangtze River. It covers a surface area of 42.30 km^2^ and an average depth of 3 m. Bao’an Lake (30°14′53″ N, 114°43′02″ E) is situated in the north-west of Daye City, with an average depth of 2 m and a surface area of 45.10 km^2^. Xiandao Lake (29°47′18″ N, 114°50′07″ E) is situated in the western part of Yangxin County, Huangshi, with an average depth of 25.70 m.

### 4.2. Determination of Water Quality

In situ measurements of DO and WT were performed using a portable multiparameter instrument (YSI EXO2, Yellow Springs, OH, USA). A Secchi disk was used to obtain Secchi depth (SD). For laboratory analyses, water samples were collected from 50 cm below the surface into sterile 1.5 L polypropylene bottles and transported under temperature-controlled conditions. TN was analyzed using the alkaline potassium persulfate digestion–UV spectrophotometric method. TP was quantified using the ammonium molybdate spectrophotometric method, ammonia nitrogen using Nessler’s reagent spectrophotometry, and the COD_Mn_ using the acidic potassium permanganate method. Chlorophyll a was extracted with 90% acetone and determined spectrophotometrically. All analytical procedures followed the standard protocols in the 4th edition of the Methods for the Monitoring and Analysis of Water and Wastewater [41].

### 4.3. Phytoplankton Collection and Identification

Water samples (1 L) were obtained using a plexiglass sampler and immediately preserved by adding 15 mL of Lugol’s iodine solution. After a 48 h sedimentation period, the overlying water was siphoned off, and the settled material was concentrated to a final volume of 30 mL [42]. The concentrate was thoroughly homogenized, and a 0.1 mL aliquot was introduced into a phytoplankton counting chamber. Species identification and cell enumeration were conducted under a microscope at 10× or 40× magnification using the field-of-view counting method; two to three replicate counts were made per sample. Counting errors were controlled within 15%, and the average value was used to calculate phytoplankton density (cells/L) [43].

To analyze the phytoplankton community structure, species diversity was estimated using the Margalef richness (*D*) (Equation (1)), Shannon–Wiener (*H*′) (Equation (2)), and Pielou evenness (*J*) indexes (Equation (3)) [44,45,46]. The corresponding calculation formulas are presented below. Taxa with a dominance index (Equation (4)) *Y* > 0.02 are identified as dominant species [47].

The calculation formulas for each parameter are as follows:

(1) D=(S−1)/ln N

(2) $$H^{\prime }=-\sum_{i=1}^{S}(n_{i}/N)\times \ln(n_{i}/N)$$

(3) J=H′/ln S

(4) $$Y=n_{i}/N\times f_{i}$$

where n*_i_* is the individual count of the *i*-th species per sample, *N* is the total number of individuals per sample, *f_i_* is the frequency of occurrence (%) of the *i*-th species, *S* is the overall phytoplankton species richness. The water quality evaluation criteria [48] are summarized in Table 4.

TLI is calculated as follows [49]:

(5) $$TLI(\sum )=\sum_{i=1}^{m}W_{j}\times TLI(j)$$

where *W_j_* denotes the weighting factor assigned to the trophic state index of the *j*-th parameter, and TLI(*j*) is the trophic state index for that parameter. The composite TLI was derived from five indicators: TN, TP, SD, Chl-a, and COD_Mn_ [50].

### 4.4. Data Analysis

The study area map was generated in ArcGIS 10.8 Microsoft Excel 2019 and GraphPad Prism 10.1.2 were used for data processing, descriptive statistics, normality and homoscedasticity tests, and one-way ANOVA with post hoc multiple comparisons.

Multivariate ordination was performed in Canoco 5.0. Detrended correspondence analysis (DCA) was first run for model selection: canonical correspondence analysis (CCA) was applied when the gradient length of the first DCA axis exceeded 4, with dominant species biomass as response variables; redundancy analysis (RDA) was adopted for gradient lengths < 3, with biomass of dominant functional groups as response variables. Both models incorporated water temperature, transparency, suspended solids, nitrogen and phosphorus nutrients, and permanganate index as explanatory variables to identify key drivers structuring phytoplankton communities. All figures were produced using Origin 2021.

## 5. Conclusions

Based on the seasonal monitoring of four lakes in the middle Yangtze River (Xiandao, Bao’an, Wanghu, and Cihu) from October 2022 to August 2023, the phytoplankton community structure and environmental drivers under different trophic conditions were analyzed using physicochemical, taxonomic, FG, and multivariate statistical methods. The main conclusions are as follows:

(1) Xiandao Lake was oligotrophic, with the highest annual average SD (3.41 m) and the lowest nutrient levels. Bao’an, Wanghu, and Cihu Lakes were mildly eutrophic, with nutrient indices peaking in summer. Wanghu Lake had the highest phosphorus load, whereas Cihu Lake had the highest nitrogen concentration.
(2) In total, 183 phytoplankton species from eight phyla, dominated by Chlorophyta (79 species), were identified. Bao’an Lake had the highest species richness (140 species), whereas Xiandao Lake had the highest diversity and a slightly polluted status. In contrast, Cihu and Wanghu Lakes had low evenness (J < 0.3) and poor community stability.
(3) Twenty-seven FGs were identified. The dominant FG succession patterns varied across the lakes, revealing a shift from S-type to R-type strategies with increasing nutrient levels.
(4) SD, SS, phosphate, and WT were the key drivers of community succession, with seasonal variations in their relative importance. Lake-specific drivers included SD for Xiandao Lake, phosphorus for Wanghu Lake, and nitrogen, SS, and COD_Mn_ for cyanobacterial dominance in Cihu and Bao’an Lakes.

## Figures and Tables

**Table 1:** Physicochemical characteristics of water in four representative lakes in the middle Yangtze River.

Environmental Factor	Bao’an Lake	Cihu Lake	Wanghu Lake	Xiandao Lake
SD (m)	0.38 ± 0.01 b	0.56 ± 0.18 b	0.54 ± 0.10 b	3.41 ± 0.54 a
SS (mg/L)	19.39 ± 4.37 a	6.45 ± 4.09 c	12.98 ± 5.05 b	1.61 ± 1.18 d
COD_Mn_ (mg/L)	5.96 ± 1.32 a	5.23 ± 2.15 a	5.21 ± 2.31 a	1.73 ± 0.29 b
TN (mg/L)	0.78 ± 0.05 b	1.14 ± 0.30 a	1.15 ± 0.69 a	0.29 ± 0.10 c
TP (mg/L)	0.07 ± 0.02 b	0.08 ± 0.02 b	0.18 ± 0.06 a	0.02 ± 0.01 c
NO_3_^−^-N (mg/L)	0.20 ± 0.10 ab	0.22 ± 0.23 a	0.07 ± 0.05 c	0.1 ± 0.1 bc
PO_4_^3−^-P (mg/L)	0.01 ± 0.01 b	0.02 ± 0.01 b	0.06 ± 0.4 a	0.01 ± 0.00 b
NH_3_-N (mg/L)	0.18 ± 0.07 b	0.26 ± 0.09 ab	0.29 ± 0.20 a	0.09 ± 0.06 c
Chl-a (μg/L)	20.13 ± 13.8 ab	41.76 ± 17.68 a	20.23 ± 15.12 ab	1.18 ± 1.22 b
TLI	55.40 ± 0.98 ab	57.13 ± 1.77 a	54.79 ± 0.88 b	23.13 ± 1.39 c

Note: Data are presented as mean ± standard deviation. Different lowercase letters in the same row indicate significant differences (*p* < 0.05). Groups sharing a common letter are not significantly different from each other.

**Table 2:** Dominant phytoplankton species and dominance indices in typical lakes of the middle Yangtze River.

Lake	Type (Species)	Dominant Species	Season			
			Spring	Summer	Autumn	Winter
Bao’an Lake	Cyanobacteria	*Oscillatoria subbrevis*	---	---	0.032	---
		*Limnothrix* sp.	---	0.074	0.027	0.135
		*Pseudoanabaena limnetica*	---	0.247	0.284	0.059
		*Cuspidothrix issatschenkoi*	---	---	0.044	---
		*Dolichospermum convolutus*	---	0.024	0.092	---
		*Dolichospermum* sp.	---	---	0.076	---
		*Aphanizomenon* sp.	---	0.28	0.251	---
		*Merismopedia minima*	0.131	---	0.039	---
		*Oscillatoria tenuis*	0.045	0.086	---	---
		*Dactylococcopsis* sp.	0.059	---	---	0.087
		*Rhabdogloea* sp.	0.023	---	---	---
		*Synechocystis minuscula*	0.12	---	---	---
	Bacillariophyta	*Cyclotella* sp.	0.121	---	---	0.197
	Chlorophyta	*Scenedesmus bijuga*	0.078	---	---	0.036
		*Scenedesmus quadricauda*	0.105	---	---	0.033
		*Pediastrum simplex*	---	---	---	0.11
		*Closterium parvulum* var. *angustum*	0.023	---	---	0.038
Wanghu Lake	Cyanobacteria	*Dolichospermum* sp.	0.831	---	---	---
		*Oscillatoria tenuis*	---	0.602	---	---
		*Oscillatoria princeps*	---	0.031	---	---
		*Oscillatoria anguina*	---	0.15	---	---
		*Spirulina platensis*	---	0.042	---	---
		*Pseudoanabaena limnetica*	---	0.024	---	---
		*Microcystis* sp.	---	0.056	---	---
		*Dolichospermum* sp.	---	---	0.022	---
		*Merismopedia minima*	---	---	0.027	---
		*Microcystis* sp.	---	---	0.725	---
		*Limnothrix* sp.	---	---	---	0.059
		*Synechocystis minuscula*	---	---	---	0.038
		*Dactylococcopsis* sp.	---	---	---	0.067
	Bacillariophyta	*Cyclotella* sp.	0.02	---	---	0.224
	Chlorophyta	*Scenedesmus quadricauda*	—	---	0.035	0.065
		*Tetrastrum staurogeniae forme*	—	---	0.021	---
		*Scenedesmus bijuga*	—	---	---	0.152
Cihu Lake	Cyanobacteria	*Limnothrix* sp.	0.029	0.363	0.097	0.08
		*Pseudoanabaena limnetica*	0.754	0.232	0.122	0.162
		*Merismopedia minima*	0.021	0.045	0.462	---
		*Oscillatoria tenuis*	---	0.024	---	---
		*Leptolyngbya* sp.	---	0.039	0.025	---
		*Raphidiopsis* sp.	---	0.058	0.028	---
		*Synechocystis minuscula*	---	0.022	---	---
		*Spirulina* sp.	---	---	0.047	0.046
		*Rhabdogloea* sp.	---	---	0.034	---
		*Merismopedia punciata*	---	---	0.029	---
		*Anabaena oscillarioides*	---	---	---	0.045
		*Dactylococcopsis* sp.	---	---	---	0.078
	Bacillariophyta	*Cyclotella* sp.	0.021	---	---	0.045
		*Synedra acus*	0.022	---	---	---
		*Hydrosera whampoensis*	---	---	---	0.024
	Chlorophyta	*Scenedesmus quadricauda*	---	---	---	0.032
		*Tetrastrum staurogeniae forme*	---	---	---	0.038
		*Tetraspora* sp.	---	---	---	0.028
Xiandao Lake	Cyanobacteria	*Synechocystis minuscula*	---	0.115	---	---
		*Chroococcus* sp.	---	0.038	---	---
		*Dactylococcopsis* sp.	---	0.296	---	---
		*Limnothrix* sp.	---	---	0.041	---
		*Pseudoanabaena limnetica*	---	---	0.268	---
		*Dolichospermum convolutus*	---	---	0.026	0.348
		*Dolichospermum* sp.	---	---	0.022	---
		*Aphanizomenon* sp.	---	---	0.157	0.24
	Bacillariophyta	*Cyclotella* sp.	0.249	0.167	---	---
		*Rhizosolenia* sp.	0.027	---	---	---
		*Synedra acus*	0.035	---	---	---
	Chlorophyta	*Scenedesmus bijuga*	0.024	0.049	---	---
		*Oocystis solitaria*	---	0.023	---	---
		*Oocystis parva*	---	0.022	---	---
		*Coelastrum reticulatum*	---	0.035	---	---
	Chrysophyceae	*Dinobryon* sp.	0.048	---	---	---
	Xanthophyceae	*Botrydiopsis* sp.	0.476	---	---	---

“---” indicates that the species was not detected in that season. Phytoplankton with a dominance value of Y > 0.02 are defined as the dominant species.

**Table 3:** Composition of phytoplankton FGs in typical lakes of the middle Yangtze River.

FGs Code	Representative Species	Habitat Description	Growth Strategy
B	*Cyclotella* sp. *Hydrosera whampoensis*	Fully mixed, mesotrophic small to medium-sized water bodies	S
D	*Synedra* sp.	Nutrient-rich, turbid water bodies	R
E	*Mallomonas* sp. *Dinobryon* sp.	Eutrophic or heterotrophic, small water bodies	S
F	*Oocystis* sp. *Tetraspora* sp. *Kirchneriella* sp.	Mesotrophic to eutrophic, clear, strongly mixed	C
H1	*Anabaena* sp. *Aphanizomenon* sp. *Dolichospermum* sp. *Cuspidothrix issatschenkoi*	Low phosphorus, eutrophic, stratified, low nitrogen, shallow water	R
J	*Chodatella* sp. *Chlorella* sp. *Tetraedron* sp. *Scenedesmus* sp. *Crucigenia* sp. *Pediastrum* sp. *Tetrastrum staurogeniaeforme* *Golenkinia* sp. *Coelastrum* sp. *Botrydiopsis* sp.	Highly eutrophic, mixed, shallow water	R
Lo	*Rhabdogloea* sp. *Synechocystis* sp. *Chroococcus* sp. *Merismopedia* sp.	Oligotrophic to eutrophic, medium to large water bodies, varying depths	C/S
MP	*Oscillatoria* sp. *Cocconeis* sp. *Surirella* sp. *Cymatopleura* sp. *Achnanthes* sp. *Gymnodinium* sp.	Frequently stirred, turbid, shallow water	R
M	*Microcystis* sp.	Small to medium-sized, eutrophic to hyper-eutrophic, stable, high transparency	R
N	*Closterium* sp. *Cosmarium* sp.	Continuous or semi-continuous mixing layers	S
P	*Closterium* sp. *Melosira* sp.	Continuous or semi-continuous mixing layers	C/R
Q	*Gonyostomum* sp.	Humic-rich, acidic, small water bodies	S
S1	*Anabaenopsis* sp. *Leptolyngbya* sp. *Limnothrix* sp.	Sensitive to light and scouring, suited to living in dark environments	R
S2	*Spirulina* sp.	Warm, highly alkaline, shallow water	R
SN	*Raphidiopsis* sp.	Warm, mixed	R
T	*Mougeotia* sp.	Continuous mixing layers	S
TB	*Navicula* sp. *Cymbella* sp.	Strong, rapid currents	R/S
TC	*Oedocladium* sp.	Eutrophic, stagnant water bodies	C
TD	*Ulothrix* sp.	Mesotrophic, stagnant water bodies with slow flow; rivers with dense populations of large emergent plants or epiphytic, multicellular charophytes, filamentous Chlorophyta and benthic Bacillariophyta	C/S
W1	*Euglena* sp. *Phacus* sp.	Organic pollution, shallow water	R
W2	*Trachelomonas* sp. *Strombomonas* sp.	Mesotrophic, shallow water	C
X1	*Ankistrodesmus* sp.	Hyper-eutrophic, shallow water	R
X2	*Chlamydomonas* sp. *Chlorogonium* sp. *Pteromonas* sp.	Moderately to highly eutrophic water bodies	R/C
X3	*Schroederia* sp.	Mixed, oligotrophic shallow water bodies	S
XPh	*Phacotus* sp.	High calcium content, good light conditions, alkaline, small water bodies	S
Y	*Cryptomonas* sp.	Still water environments	C
Z	*Synechocystis* sp.	Low nutrient levels, low light conditions	S

**Table 4:** Water quality testing standards.

Methodology	Scope	Water-Quality Category
Margalef richness index (D)	0–1	severe pollution
	1–3	medium pollution
	>3	light or no pollution
Shannon–Weiner index (H′)	0–1	severe pollution
	1–3	medium pollution
	>3	light or no pollution
Pielou’s evenness index (J)	0–0.3	severe pollution
	0.3–0.5	medium pollution
	>0.5	light or no pollution

## Data Availability

The original contributions presented in this study are included in the article; further inquiries can be directed to the corresponding author.

## Supplementary Materials

The following supporting information can be downloaded at: https://www.mdpi.com/article/10.3390/plants15142108/s1.
